# Effect of Aqueous Stem Bark Extract of *Khaya senegalensis* on Some Biochemical, Haematological, and Histopathological Parameters of Rats

**DOI:** 10.1155/2013/803835

**Published:** 2013-11-18

**Authors:** A. Onu, Y. Saidu, M. J. Ladan, L. S. Bilbis, A. A. Aliero, S. M. Sahabi

**Affiliations:** ^1^Department of Biochemistry, Usmanu Danfodiyo University, PMB. 2346, Sokoto, Nigeria; ^2^Department of Biological Sciences, Usmanu Danfodiyo University, PMB. 2346, Sokoto, Nigeria; ^3^Department of Histopathology, Usmanu Danfodiyo University, PMB. 2346, Sokoto, Nigeria

## Abstract

The subchronic effect of aqueous stem bark extract of *Khaya senegalensis* on some biochemical, haematological, and histopathological parameters of rats was investigated. The rats were divided into six groups of five rats per group. Groups I to VI were administered graded doses of 0, 400, 800, 1200, 1600, and 2000 mg/kg bw, respectively. The result of study revealed that administration of the *Khaya senegalensis* for twenty-eight days at the experimental dose resulted in significant (*P* < 0.05) increase in urea, electrolytes (Na^+^, K^+^), and creatinine levels. The extract also significantly (*P* < 0.05) increased serum activity of ALT, AST, and ALP. The levels of protein, albumin, and bilirubin were significantly changed when compared to their control values, but they were not dose dependent. The hematological indices assayed in this study were not significantly affected at the experimental dose when compared to the control values. Histological studies of the liver showed cellular degeneration and necrosis and bile duct hyperplasia and fibrosis with lymphocytic infiltration of the hepatocyte, providing supportive evidence for discussing the biochemical findings, indicative of functional derangement. The histological architecture of the kidney and that of the heart were however preserved. The result of this study indicates that the aqueous stem bark extract of *K. senegalensis* may affect the cellular integrity of vital organs of the body.

## 1. Introduction

The therapeutic value of medicinal plants has long been exploited for the management of various disease conditions in traditional practice. This practice has gained appreciable acceptance in health care delivery in developing and developed nations with the notion that they are relatively harmless, but research is beginning to show that some of them may be toxic.

*Khaya senegalensis* (Desr.) A. Juss (Family: Meliaceae) is a tree that is widely distributed in the sub-Saharan savannah from Senegal to Sudan and Uganda. It is a round evergreen crown of dark shiny foliage, pinnate leaves, and characteristic round capsules that grows up to 40 m high [1]. The therapeutic value of *Khaya senegalensis* has been recognized in different systems of traditional medicine for the treatment of various conditions. The decoction of the stem bark extract is commonly used for treating jaundice, dermatoses, malaria, fever, mucous diarrhea, and venereal diseases as well as for hookworm infection and a taeniacide remedy [2, 3]. *Khaya senegalensis* extracts have been reported to exhibit anti-inflammatory effects [4] as well as anti-bacterial [5], antihelmintic [6], antitumor, antioxidant [7] and antiplasmodial activities [8]. The stem bark extract and the chemical constituent profile have been the subject of extensive phytochemical and pharmacological investigations since the 1960s [9–11]. Despite the extensive research on the stem bark extract of *Khaya senegalensis*, a dearth of information on the toxicological profile of this plant still exists. This work is therefore designed to evaluate the acute and subchronic toxicity effects of the extract on the liver and kidney.

## 2. Materials and Methods

### 2.1. Plant Materials

Stem bark of *K. senegalensis *was collected from the main campus of Usmanu Danfodiyo University, Sokoto, (130 21′ 16′′ N and 50 5′ 37′′ E) in the month of June 2011. The plant was identified by a taxonomist, Mal. A.M. Umar from the Botany Unit, Department of Biological Sciences, Usmanu Danfodiyo University, Sokoto, and a voucher specimen (UDUS/VS/2011/21) was prepared and deposited in the herbarium of the same department.

### 2.2. Experimental Animals

Albino male rats weighing 150–200 g were purchased from the Animal House, Usmanu Danfodiyo University, Sokoto, Nigeria. They were housed in metal cages and allowed free access to a standard laboratory pellet diet (ECWA Feed Nig Ltd.) and water *ad libitum *under a 12-hour light-dark cycle throughout the experimental period.

### 2.3. Preparation and Extraction of Plant Samples

Plant materials were washed and air-dried at room temperature until a constant weight was obtained. The samples were ground using a pestle and mortar and the powdered samples were then bagged and stored in plastic bags until required.

The plant sample was separately placed in an Erlenmeyer flask and one liter of distilled water was added into the flask and it was allowed to soak for a minimum of 24 hours. 5% w/v of the plant material was prepared. The mixture was filtered using a piece of clean, sterile Muslin cloth to remove debris and then filtered on Whatman filter paper. The resultant filtrate was evaporated to dryness [12] and the percentage recovery was calculated (13.3%).

### 2.4. Acute Toxicity Study

Acute oral toxicity was performed using the up- and down-procedure of the Organisation for Economic Co-operation and Development [13]. This procedure involves the limit and main test, which was also used for the selection of a starting dose as well as determination of LD_50_ of the testing material.

### 2.5. Subchronic Toxicity Study

Animals were divided into six groups of five animals each. The test substance was administered orally to the rats in groups II–IV in a gradation of 400, 800, 1200, 1600, and 2000 mg/kg body weight per day for 28 days, while group I (control group) animals were given an equivalent volume of normal saline orally for the same period of 28 days under the same conditions.

The body weight changes were monitored weekly throughout the experimental period. On the 29th day, after an overnight fast, the rats were anaesthetized in a chloroform vapour and blood samples were collected from the animals by cardiac puncture into labeled Vacutainers for biochemical and haematological assays. Subsequently, the animals were sacrificed by decapitation; the organs (kidney, liver, and heart) were removed, weighed, and then fixed in 10% formalin for histopathological examinations [13].

### 2.6. Biochemical and Haematological Analysis

Serum total protein was determined using the biuret method; cupric ions in the biuret reagent react with the peptide bonds of the protein molecule in an alkaline solution to form a blue colored complex [14]. Albumin was determined using the Bromocresol Green method; albumin binds with Bromocresol Green in an acidic medium to form a blue-green complex whose colour is proportional to the concentration of albumin in the serum [15]. Transaminase activity (aspartate aminotransferase (AST) and alanine, aminotransferase (ALT)) was determined using the Reitman and Frankel [16] method; ALT was measured by monitoring the concentration of pyruvated hydrazone that is formed with 2,4-dinitrophenylhydrazine while AST was measured by monitoring the concentration of oxaloacetate hydrazone that is formed with 2,4-dinitrophenylhydrazine. Alkaline phosphatase (ALP) activity was determined using the colorimetric method of Rec [17]; the hydrolysis of 4-nitrophenyl phosphate at alkaline pH to 4-nitrophenol and inorganic phosphate is catalysed by alkaline phosphatase and the intensity of the colour is directly proportional to the activity of ALP. Urea was investigated using the Urease-Berthelot colorimetric method of Fawcett and Scott [18]; urea in serum is hydrolysed to ammonia in the presence of urease. The ammonia formed is measured spectrophotometrically by the Berthelot reaction. Creatinine was measured by the picrate method [19]; creatinine reacts with picric acid in an alkaline medium to form a yellow-red colored complex which can be measured spectrophotometrically at 520 nm. Serum electrolytes Na^+^ and K^+^ were measured using a flame emission spectrophotometer; Na^+^ and K^+^ are atomised using a flame; the amount of atomisation is proportional to the quantity of the element in the solution, while HCO_3_ was estimated using a titrimetric method [20]. Packed cell volume (PCV) of samples was determined using microhematocrit centrifuge at 12000 g for 5 min [21]; white blood cell (WBC) count and differentials were determined by mechanical expansion and optical magnification, augmented by supravital cell staining [21].

### 2.7. Histopathological Analysis

Three animals from each group were selected randomly and dissected through a central abdominal incision. The kidney, heart, and liver samples were collected and immediately fixed in 10% formal saline in labeled sample plastic bottles. The tissues were dehydrated in graded concentrations of xylene, embedded in molten paraffin wax, and sectioned at 5 *μ*. Tissue sections were fixed on glass slides and stained with hematoxylin and eosin for light microscopy at 400x [22]. Photomicrographs of some of the tissues were taken using a microscope fitted with a camera unit.

### 2.8. Statistical Analysis

Data were expressed as mean ± standard deviation for the given number of observations. The results were analyzed using one-way analysis of variance (ANOVA), followed by Dunnett's multiple comparison test using GraphPad Instat Software. Differences were considered significant when *P* < 0.05.

## 3. Results

### 3.1. Acute Toxicity Study

**Figure 1:**
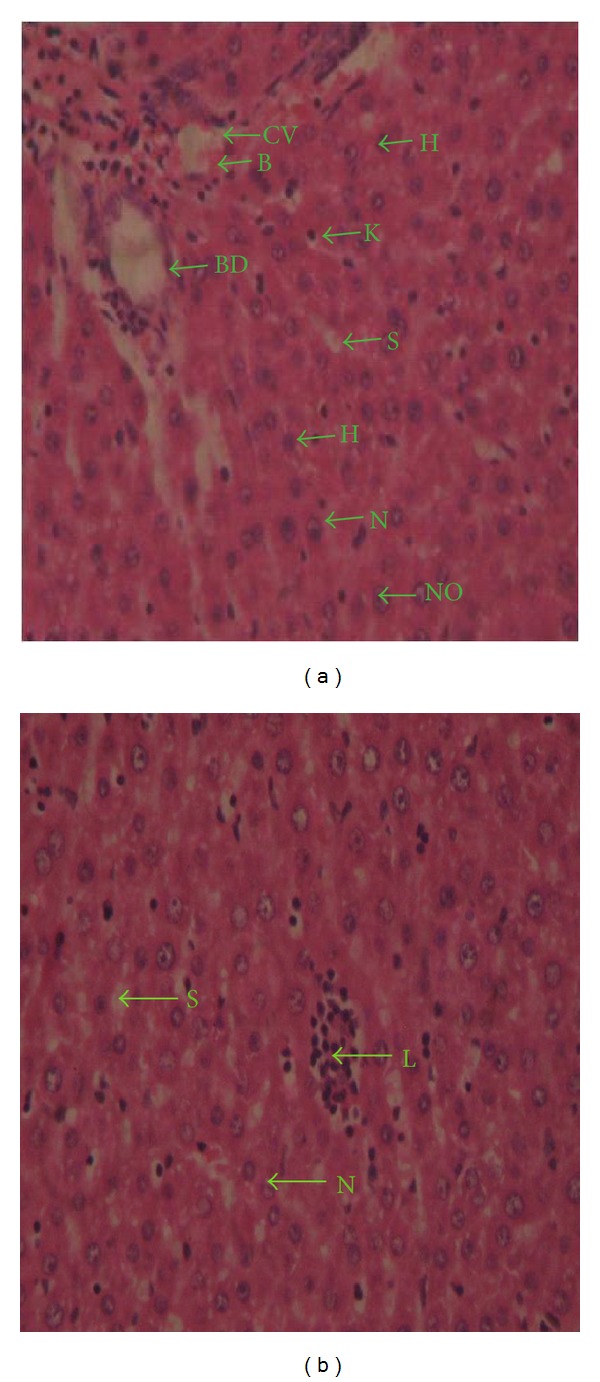
Photomicrographs of liver sections from control dose group (a) and treated dose group (b), H&E stain, 400x. (a) Normal architecture of the liver showing the bile duct (BD) and central vein (CV) in the center of hepatic lobule filled with blood (B). Hepatocytes (H), arranged in the form of cords, are rounded in a polyhedral shape and radiate peripherally; they show the nucleus (N) with clear nuclear membrane and nucleolus (No), and cords are separated by sinusoids (S) with Kupffer cells (k). (b) Normal architecture of the liver that show the nucleus (N) with a clear nuclear membrane, and cords are separated by sinusoids (S) with Kupffer cells (k), with lymphocytes (L) surrounding the central vein.

The LD_50_ was found to be greater than 5000 mg/kg body weight. During the course of the toxicity experiment, the animals were observed for any change in behavioural pattern, physical characteristics, and activity. The rats in all the steps displayed no appreciable change in physical or behavioural activities; they fed well and moved about in their cages well. The histological preparations of the liver after treatment with the extract are presented in Figures 1(a) and 1(b); both the normal and experimental preparations appear within the range of a preserved histological architecture.

### 3.2. Effect of Subchronic Oral Administration of Aqueous Stem Bark Extract of *K. senegalensis* on Ratio of Organ Weight : Body Weight of Rats and Percentage Change in Body Weight of the First Day and Last Day of the Experimental Period

The effects of oral administration of aqueous extract of *Khaya senegalensis *stem bark on the ratio of organ weight** **:** **body weight of experimental rats and percentage change in body weight of the first and last days of the experimental period are shown in Table 1. Prolonged administration of the extract for 28 days resulted in significant (*P* < 0.05) decrease in percentage change in body weight of the experimental rats in a dose dependent manner when compared to the values of the control group. Administration of several doses of the extract to their corresponding groups had no significant (*P* > 0.05) effect on the ratio of kidney weight** **:** **body weight as well as ratio of heart weight** **:** **body weight when compared to the values of their corresponding control group. However the ratios of liver weight** **:** **body weight and spleen weight** **:** **body weight were observed to be significantly different at the higher doses of 1600 mg/kg (*P* < 0.05) and 2000 mg/kg (*P* < 0.01) when compared to their corresponding control values.

### 3.3. Effect of Subchronic Oral Administration of Aqueous Stem Bark Extract of *K. senegalensis *on Kidney Function Parameters of Rats

The effect of aqueous stem bark extract of *K. senegalensis *on serum urea, creatinine, potassium ion, and sodium ion is shown in Table 2. The administration of the extract increased the serum concentration of urea and potassium ion significantly (*P* < 0.05) in all the different dose groups when compared to their corresponding control values. There was an observed corresponding increase in the values of creatinine and sodium levels in all the dose groups, with significant (*P* < 0.05) difference at the 1600 mg/kg and 2000 mg/kg body weight dose group.

### 3.4. Effect of Subchronic Oral Administration of Aqueous Stem Bark Extract of *K. senegalensis *on Liver Function Parameters of Rats

 The effect of subchronic oral administration of aqueous stem bark extract on serum total protein, albumin, globulin, albumin/globulin ratio (A : G), some transaminases activities, alkaline phosphatase activity, and bilirubin is presented in Table 3. The results indicated a significant (*P* < 0.05) reduction in the values of total protein and albumin in a dose dependent manner. The observed significant (*P* < 0.05) effect on globulin and albumin/globulin ratio was however not dose dependent. The effect of the extract resulted in a significant (*P* < 0.05) increase in AST, ALT, and ALP activities when compared to their corresponding control values. Similarly, the extract exerted a significant (*P* < 0.05) decrease in total bilirubin, conjugated bilirubin, and unconjugated bilirubin when compared to their corresponding control values.

### 3.5. Effect of Subchronic Oral Administration of Aqueous Stem Bark Extract of *K. senegalensis* on Haematological Indices of Albino Rats

Results on the effect of sub-chronic oral administration of aqueous stem bark extract of *K. senegalensis *on haematological indices are presented in Table 4. The results demonstrate that the extract at the different experimental doses had no significant (*P* > 0.05) effect on the haematological indices.

### 3.6. Histopathology

The livers of animals in the control group (0 mg/kg) showed typical hepatolobular architecture, consisting of a central vein with radiating cords of hepatocytes separated by sinusoids; portal areas composed of the portal vein, hepatic artery, and bile duct were situated at the periphery. The hepatocytes were polygonal in shape, with central, lightly stained nucleus and clear nucleolus; few binucleated cells were also present, and the cytoplasm was regularly distributed without vacuolations. In the treated dose groups (400, 800, 1200, 1600, and 2000 mg/kg bw) the general hepatolobular architecture of the liver was deranged; there was loss of radial arrangement of hepatocytes and sinusoids; the number of binucleated cells was increased; inflammatory cells, especially lymphocytes, were found infiltrating around the portal track; areas of necrosis were found around the central vein.

**Figure 2:**
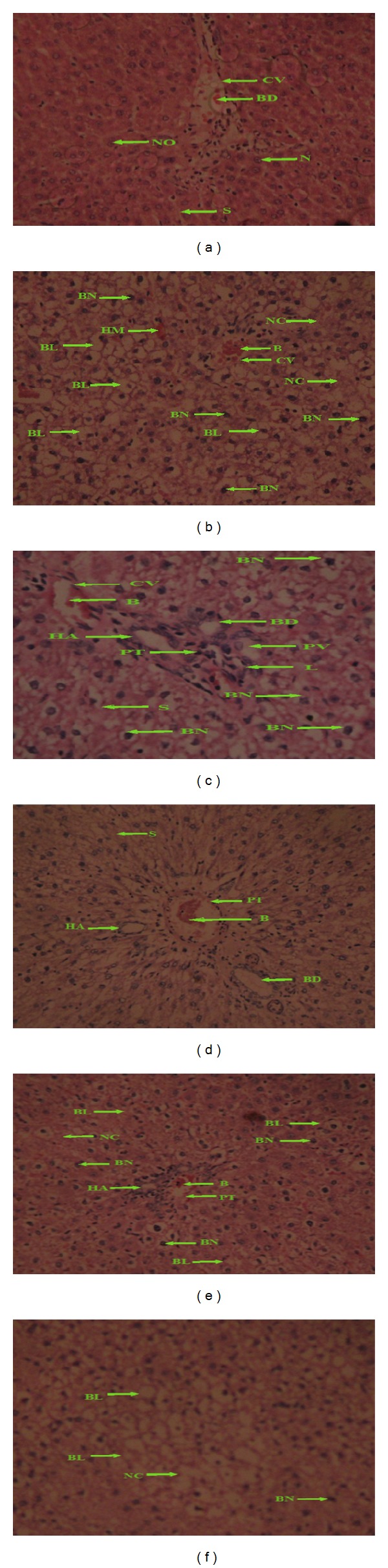
Photomicrographs of liver sections, H&E stain 400x. (a) 0 mg/kg bw: normal architecture of the liver: central vein (CV) in the center of the hepatic lobule is filled with blood (B). Hepatocytes, arranged in the form of cords, are rounded in a polyhedral shape and radiate peripherally; they show the nucleus with a clear nuclear membrane and nucleolus (NO); cords are separated by sinusoids (S) with Kupffer cells. (b) 400 mg/kg bw: deranged architecture of the liver, extensive ballooning (BL) of hepatocytes with hyperchromatic nuclei, and nucleoli are not clearly seen; multiple binucleated cells (BN) and sinusoidal arrangement are also deranged, observed centrilobular necrosis (NC). (c) 800 mg/kg bw: distorted architecture of the liver with ballooning degeneration of hepatocytes (H). Observed hyperchromatic nuclei (N) and nucleoli are not clearly seen; multiple binucleated cells (BN) and sinusoidal (S) arrangement also seems to have been deranged. Periportal (PT) inflammation consisting of collection of lymphocytes (L) around the bile duct (BD), portal vein (PV), and hepatic artery (HA). (d) 1200 mg/kg bw: feathery ballooning of hepatocytes with sinusoidal (S) arrangement being deranged; portal triaditis (PT) showing inflammation consisting of a collection of lymphocytes around the bile duct (BD). (e) 1600 mg/kg bw: deranged architecture of liver with hepatocyte vacuolation, ballooning (BL) of hepatocytes with nucleoli not clearly seen, multiple binucleated cells (BN) and sinusoidal arrangement have been deranged; periportal triaditis (PT) showing distorted triads and inflammation consisting of collection of lymphocytes (L) around the hepatic artery (HA), observed hepatic necrosis (NC). (f) 2000 mg/kg: extensive ballooning (BL) of hepatocytes with multiple binucleated dilation with centrilobular necrosis (NC).

Examination of the liver histological preparations obtained from all the experimental treated groups showed comparable changes as observed in Figures 2(a) to 2(f).

**Figure 3:**
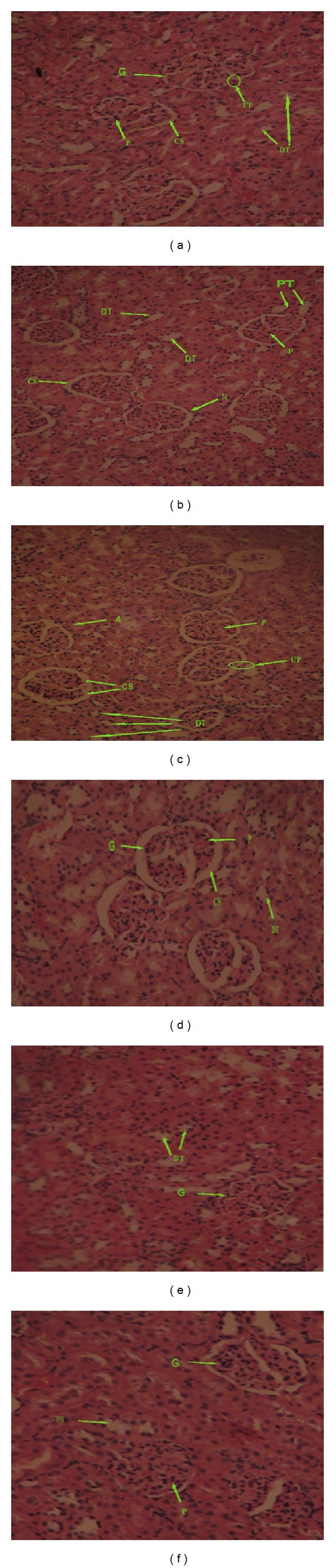
Photomicrographs of kidney sections, H&E stain 400x. (a) 0 mg/kg bw: observed normal proximal and distal tubules (DT), glumerulus (G), blood vessels, and interstition. Podocyte (P), urinary pole (UP), and corpuscular space (CS) appear within normal range. (b) 400 mg/kg bw: proximal (PT) and distal tubule (DT), glomerulus (G), blood vessels, and interstition. Podocyte (P) and corpuscular space (CS) appear within normal range. (c) 800 mg/kg bw: normal architecture of a kidney section; cross-section of an arteriol (A) and a closely packed renal tubule is observed. (d) 1200 mg/kg bw: normal distal tubule (DT), glomerulus (G), blood vessels and interstation. (e) 1600 mg/kg bw: a closely packed renal tubule is observed, and corpuscular space (CS) appears within normal range. (f) 2000 mg/kg bw: normal proximal tubule (PT), glomerulus (G), and corpuscular space (CS) appear within normal range.

**Figure 4:**
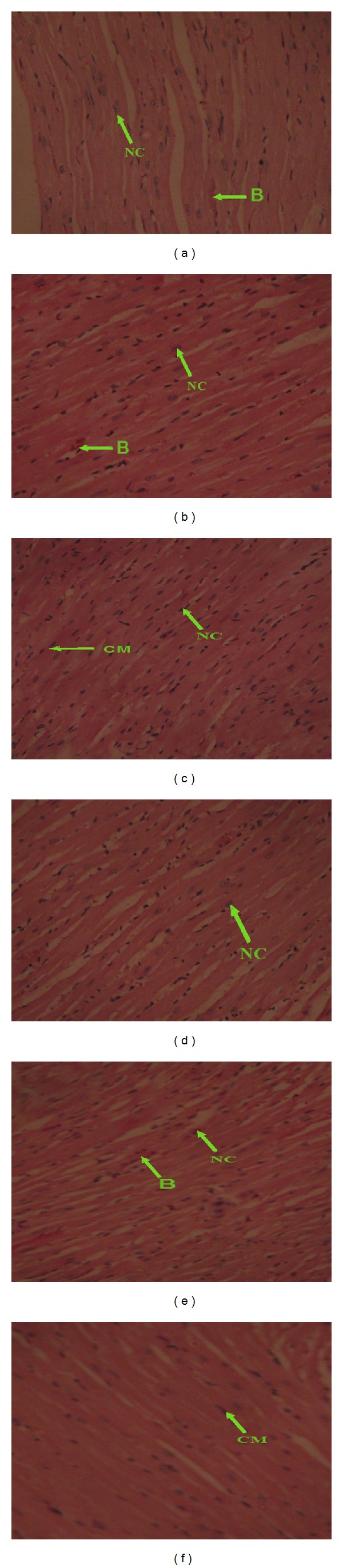
Photomicrographs of heart sections, H&E stain 400x. (a) 0 mg/kg bw: normal architecture of the heart section. A cardiac myocyte (CM) appears within the normal limits with nuclei (NC) present. A normal intercalation disk, with mild congestion. (b) 400 mg/kg bw: the heart section appears within normal limits with visible cardiac myocyte and nuclei (NC), observed normal intercalation disk, with mild congestion. (c) 800 mg/kg bw: normal architecture of the heart section. A cardiac myocyte (CM) appears within the normal limits with nuclei present (NC). (d) 1200 mg/kg bw: heart sections: cardiac myocyte, nuclei (NC), and intercalation disk appear within the normal limits. (e) 1600 mg/kg bw: normal architecture of the heart section. (f) 2000 mg/kg bw: normal architecture of the hearth section.

The histological preparations of the kidney and heart of the rats based on the subchronic toxicity study of aqueous stem bark extract of *K. senegalensis *indicated that they had normal architecture (Figures 3 and 4). The observed changes, however, in all the treated groups were restricted to only mild congestion and were not dose dependent.

## 4. Discussion

The LD_50_ of aqueous stem bark extract of *K. senegalensis* was found to be greater than 5000 mg/kg; it may therefore be considered nontoxic; although this does not predict the lethal dose in humans, it however provides a guide for choosing the dose for use in sub-chronic studies.

Body weight is normally investigated as a sensitive indicator of chemically induced changes to organs. The comparison between the ratiometric differences of organ : body weight of the control group and the treated groups has been used to evaluate the toxic effect of the test substance [23]. The result of this study shows that sub-chronic administration of *K. senegalensis *does not affect the anatomical proportion (ratios of kidney : body weight and heart : body weight) as compared to normal control; this may be that the defensive mechanism of the animal has not been overcome and/or may be that the dose has not accumulated sufficiently to manifest any significant change. However, the significant increase of the ratio of liver : body weight (hepatomegaly) following the administration of the extract may be attributed to tissue oedema (oedematous) and ballooning resulting in necrosis; this may be due to the role of the liver in detoxification and excretion. The spleen at the dose group of 2000 mg/kg body weight was significantly (*P* < 0.05) increased (splenomegaly): this could result from any one of the following: hemolytic anemia, cirrhosis, hepatic vein obstruction, and portal vein obstruction. It could also have been a result of an induced alpha-mannosidosis by the extract, that is, if the extract was able to penetrate into the lysosome. It is therefore worthy to mention that the higher dose levels (1600 and 2000 mg/kg body weight) should be used with caution.

The possibility of renal insufficiency or dysfunction occurring as a result of the toxic effect of the extract may be eminent. The observed (Table 2) significant increase in serum sodium ions with a concomitant significant increase in potassium ion levels may be due to renal dysfunction resulting from the inability of the kidney to regulate an electrolyte balance. The extract may also interact with specific hormone receptors resulting in increased production of aldosterone and mineralocorticoids which may be responsible for the observed hypernatraemia. Furthermore, the marked elevation in serum urea level in this study may suggest that the extract caused renal impairment in the rats or it may be a result of increased protein catabolism. However since urea is not a complete estimation of renal function, the observed significant increase in creatinine may further buttress the extract as toxic to the kidney at the doses used in this study.

Serums ALT and AST considered in this study are important and play significant role in the diagnosis of liver cytolysis [24]. The estimation of the levels of protein and bilirubin is used to examine the synthetic and excretory function of the liver, respectively. Measurement of the activities of enzymes in tissues and body fluids plays a significant and well-known role in disease investigation and diagnosis [25]. Tissue enzyme assay can also indicate tissue cellular damage long before structural damage can be picked up by conventional histological techniques. Such measurement can also give an insight to the site of cellular tissue damage as a result of assault by the plant extract [26].

In this study, the resultant increase in the activities of serums ALT and AST may be an indication of liver cytolysis, through which they are released into circulation. ALT is located in the cytosol of hepatocytes and the enzyme is considered a more sensitive marker of hepatocellular damage than AST. AST is found in the cytoplasm and mitochondria in different tissues of the heart, liver, kidney, skeletal muscle, pancreas, and erythrocyte [27]. The aminotransferases are significant in amino acid metabolism as they help in retaining amino groups during the degradation of amino acids which are further used for the synthesis of new amino acids, and hence they are involved in the biochemical regulation of the intracellular amino acid pool. Also, when these enzymes are mobilized from the liver into the free circulation, glutamate concentration may be affected with a resultant decrease in glutathione, one-third of which is formed from glutamate [28]. Detoxification is the essential role of glutathione, and cells are exposed to an attack by the active metabolite when it is depleted. Also, a significant (*P* < 0.05) change in the level of total bilirubin was observed (Table 2). This may indicate that the functional integrity of the liver has been affected by the damage caused to the liver by the plant extract [29]. Bilirubin is assayed to measure the binding, excreting, and conjugating ability of a hepatocyte. Hence, the observed increase may indicate that a component of the plant extract, might have competed and displaced the binding of bilirubin on albumin or the uptake of bilirubin is inhibited by the plant extract; this may also account for the increased level of unconjugated bilirubin. All these would lead to an increase in total bilirubin in the serum. Extract administration produced a significant decrease in the total protein concentration in all the dose groups with the exception of 400 mg/kg body weight. Hence the aqueous stem bark extract might inhibit the synthesis of some proteins, thereby resulting in the decrease in serum total protein. It has been reported that low protein level results when there is extensive liver damage [30]. The result of this study revealed significant (*P* < 0.05) increase in the activity of serum alkaline phosphatase (ALP). ALP is often employed to assess the integrity of the plasma membrane of the liver [31]. The significant increase in the serum ALP following administration of the plant extracts may be due to disruption of the liver plasma membrane. The result of this study therefore demonstrates that *K. senegalensis *at the experimental doses is nephrotoxic and should be used with caution when administered over a long period of time.

The possibility of the extract giving rise to haemopoietic damage, malignancy, allergy, or compromise to the immune status could be ruled out since all haematological parameters assayed did not show any significant changes when compared with their corresponding controls.

The liver being an organ of detoxification is the first organ that encounters all absorbed materials from the gastrointestinal tract: it has been shown to respond to toxicological insults in a number of ways including cellular degeneration and necrosis and bile duct hyperplasia and fibrosis [32]. These findings are consistent with those of Garba et al. [33] and Mutalik et al. [34]; its protective property by detoxification may explain the preserved architecture of the kidney and heart. The mild to moderate congestion as well as hemorrhage observed on the histological slides could be attributed to surgical handling of the organs since these conditions were also observed in the normal groups.

## 5. Conclusion

These observations suggest that long term administration of* K. senegalensis* may be toxic and its toxicity may be organ specific.

## Figures and Tables

**Table 1:** Effect of subchronic oral administration of aqueous stem bark extract of *K. senegalensis* on ratio of organ weight : body weight of rats and percentage change in body weight of the first day and last day of the experimental period.

Dose mg/kg bw	Kidney : body weight	Liver : body weight	Heart : body weight	Spleen : body weight	Change in body weight (%)
0	0.0053 ± 0.0001	0.0348 ± 0.0017	0.0028 ± 0.0005	0.0026 ± 0.0001	62.64 ± 8.5
400	0.0057 ± 0.0003	0.0365 ± 0.0016	0.0029 ± 0.0008	0.0028 ± 0.0004	47.11 ± 4.8^b^
800	0.0051 ± 0.0002	0.0365 ± 0.0013	0.0030 ± 0.0006	0.0030 ± 0.0003	43.39 ± 13.1^b^
1200	0.0052 ± 0.0003	0.0366 ± 0.0016	0.0030 ± 0.0006	0.0033 ± 0.0005	41.84 ± 3.4^b^
1600	0.0057 ± 0.0003	0.0383 ± 00.0012^b^	0.0031 ± 0.0004	0.0034 ± 0.0005	37.45 ± 4.8^b^
2000	0.0055 ± 0.0004	0.0398 ± 0.0017^b^	0.0031 ± 0.0005	0.0043 ± 0.0001^b^	35.32 ± 9.9^b^

Values are expressed as mean ± SD; *n* = 5. The level of significance was set at ^b^ *P* < 0.05 when compared with control at 0 mg/kg body weight, Dunnett's multiple comparison test.

**Table 2:** Effect of subchronic oral administration of aqueous stem bark extracts of *K. senegalensis* on kidney function parameters of rats.

Dose mg/kg bw	Urea (mmol/L)	Creatinine	Na^+^ (mmol/L)	K^+^ (mmol/L)
0	3.83 ± 0.38	14.47 ± 1.74	134.3 ± 1.48	4.38 ± 0.08
400	4.87 ± 0.69^b^	13.45 ± 3.02	135.5 ± 1.96	4.48 ± 0.08^b^
800	4.92 ± 0.29^b^	22.74 ± 3.46	136.9 ± 1.86	5.16 ± 0.24^b^
1200	4.89 ± 0.37^b^	29.11 ± 3.10	136.8 ± 2.38	5.48 ± 0.30^b^
1600	5.07 ± 0.16^b^	49.44 ± 9.42^b^	140.8 ± 4.55^b^	5.58 ± 0.41^b^
2000	5.02 ± 0.20^b^	70.46 ± 10.99^b^	142.8 ± 4.10^b^	6.21 ± 0.24^b^

Values are expressed as mean ± SD; *n* = 5. The level of significance was set at ^b^ *P* < 0.05 when compared with control at 0 mg/kg body weight, Dunnett's multiple comparison test.

**Table 3:** Effect of subchronic oral administration of aqueous stem bark extract of *K. senegalensis* on liver function parameters of Albino rats.

Test	0 mg/kg bw	400 mg/kg bw	800 mg/kg bw	1200 mg/kg bw	1600 mg/kg bw	2000 mg/kg bw
T. protein (g/dL)	9.56 ± 0.64	9.09 ± 0.22	8.30 ± 0.35^b^	8.04 ± 0.22^b^	7.98 ± 0.18^b^	7.59 ± 0.36^b^
Albumin (g/dL)	5.31 ± 0.44	4.08 ± 0.21^b^	4.05 ± 0.21^b^	3.99 ± 0.33^b^	4.24 ± 0.22^b^	4.35 ± 0.30^b^
Globulin (g/dL)	4.16 ± 0.23	4.85 ± 0.24^b^	4.25 ± 0.22	4.08 ± 0.17	3.70 ± 0.21^b^	3.09 ± 0.29^b^
A : G ratio	1.32 ± 0.22	0.89 ± 0.10^b^	0.96 ± 0.14^b^	0.98 ± 0.10^b^	1.12 ± 0.12	1.49 ± 0.16
AST(U/I)	52.44 ± 2.50	54.15 ± 2.87	59.60 ± 2.60^b^	58.07 ± 3.35^b^	61.24 ± 3.61^b^	84.06 ± 3.63^b^
ALT (U/I)	32.54 ± 2.50	37.58 ± 3.26	44.17 ± 3.10^b^	51.53 ± 3.00^b^	53.22 ± 2.83^b^	60.18 ± 3.40^b^
ALP (U/I)	124.74 ± 8.72	126.41 ± 12.56	135.42 ± 12.25^b^	149.43 ± 6.80^b^	198.40 ± 2.60^b^	287.93 ± 51.80^b^
T. BlL (mg/dL)	1.17 ± 0.61	1.92 ± 0.89	2.13 ± 0.71^b^	1.72 ± 0.91^b^	3.96 ± 0.60^b^	4.26 ± 0.49^b^
C. BIL (mg/dL)	0.91 ± 0.36	0.37 ± 0.21^b^	0.76 ± 0.29^b^	0.42 ± 0.17^b^	1.40 ± 0.29^b^	1.47 ± 0.27^b^
U. BIL (mg/dL)	0.26 ± 0.25	1.46 ± 0.36^b^	1.36 ± 0.32^b^	1.29 ± 0.43^b^	2.45 ± 0.47^b^	2.88 ± 0.56^b^

Values are expressed as mean ± SD; *n* = 5. The level of significance was set at ^b^ *P* < 0.05 when compared with control at 0 mg/kg body weight, Dunnett's multiple comparison test.

**Table 4:** Effect of subchronic administration of aqueous stem bark extract of *K. senegalensis* on haematological indices of Albino rats.

Dose mg/kg body weight	PCV (%)	WBC (×10^9^ L)	Neutrophils (%)	Lymphocytes (%)	Eosinophils (%)	Monocytes (%)	Platelets (×10^9 ^L)
0	41.67 ± 2.50	3.80 ± 0.76	47.67 ± 2.52	47.67 ± 3.06	3.33 ± 2.52	2.33 ± 1.53	106.67 ± 5.77
400	42.2 ± 6.08	1.04 ± 3.00	46.33 ± 6.03	49.67 ± 6.51	3.00 ± 1.00	1.33 ± 0.58	110.00 ± 10.00
800	30.00 ± 5.40	2.50 ± 0.58	44.50 ± 6.46	51.75 ± 2.36	2.50 ± 3.00	1.75 ± 0.96	115.00 ± 19.15
1200	39.50 ± 6.36	4.00 ± 2.12	45.5 ± 7.78	49.50 ± 6.36	4.50 ± 2.12	1.50 ± 0.71	130.00 ± 28.28
1600	34.33 ± 12.66	1.32 ± 3.00	48.00 ± 3.61	49.00 ± 3.61	2.67 ± 1.53	1.67 ± 1.16	130.00 ± 20.00
2000	41.67 ± 1.53	2.83 ± 3.00	52.00 ± 6.08	44.00 ± 5.29	3.33 ± 1.50	1.33 ± 0.58	110.00 ± 10.00

Values are expressed as mean ± SD; *n* = 5. The level of significance was set at *P* < 0.01 when compared with control at 0 mg/kg body weight, Dunnett's multiple comparison test.

PCV: packed cell volume; WBC: white blood cell.
